# Estrogen and Estrogen Receptor Modulators: Potential Therapeutic Strategies for COVID-19 and Breast Cancer

**DOI:** 10.3389/fendo.2022.829879

**Published:** 2022-03-23

**Authors:** Shuying Hu, Feiying Yin, Litao Nie, Yuqin Wang, Jian Qin, Jian Chen

**Affiliations:** ^1^ Guangxi Health Commission Key Laboratory of Tumor Immunology and Receptor-Targeted Drug Basic Research, Guilin Medical University, Guilin, China; ^2^ Laboratory of Environmental Pollution and Integrative Omics, Guilin Medical University, Guilin, China; ^3^ Department of Radiotherapy III, Clinical Oncology Canter, The People’s Hospital of Guangxi Zhuang Autonomous Region, Nanning, China; ^4^ Breast Center, the Second Affiliated Hospital of Guilin Medical University, Guilin, China

**Keywords:** COVID-19, selective estrogen receptor modulators, estrogen receptor, breast cancer, review

## Abstract

Owing to the ongoing coronavirus disease 2019 (COVID-19) pandemic, we need to pay a particular focus on the impact of coronavirus infection on breast cancer patients. Approximately 70% of breast cancer patients express estrogen receptor (ER), and intervention therapy for ER has been the primary treatment strategy to prevent the development and metastasis of breast cancer. Recent studies have suggested that selective estrogen receptor modulators (SERMs) are a potential therapeutic strategy for COVID-19. With its anti-ER and anti-viral combined functions, SERMs may be an effective treatment for COVID-19 in patients with breast cancer. In this review, we explore the latent effect of SERMs, especially tamoxifen, and the mechanism between ER and virus susceptibility.

## Introduction

The coronavirus disease 2019 (COVID-19), a type of acute respiratory distress syndrome (ARDS), pandemic is caused by the severe acute respiratory syndrome coronavirus 2 (SARS-CoV-2) (1). Angiotensin-converting enzyme 2 (ACE2) and transmembrane protease serine 2 (TMPRSS2) are two crucial proteins that SARS-CoV-2 uses to invade the human body. The host cell entry of SARS-CoV-2 depends on the binding of the viral spike (S) proteins to ACE2 receptors and S protein priming by TMPRSS2 (2).In physiological condition, ACE2 *via* its carboxypeptidase activity generates Angiotensin 1-9 and Angiotensin 1-7 (Ang 1–9 and Ang 1–7) and plays a critical role in the renin-angiotensin system (RAS) (3). The renin-angiotensin system (RAS) is a complicated network of G-protein coupled receptors (GPCRs) regulating many aspects of cardiovascular, pulmonary, and immune system physiology (4). The downregulation of ACE2 and the loss of catalytic activity of ACE2 in the RAS system after being engaged by the spike protein of the SARS-CoV-2, which results in ACE2 cannot transform any more Angiotensin II (AngII) into Ang1–7. Therefore, the imbalance RAS leads to the systemic pathognomonic features of patients with COVID-19 (5). Dipeptidyl peptidase 4 (DPP4), also known as CD26, which is widely distributed in various cells such as lung epithelium, endothelial, lymphocyte, and immune cells. DPP-IV plays an important role in regulating cardiovascular physiology, immune response and glucose homeostasis. DDP4 inhibitors are widely recognized as drugs for the treatment of type 2 diabetes mellitus (T2DM) (6, 7). Recently, a meta-analysis showed that DPP-IV inhibitors reduce mortality in patients with COVID-19 (8). Interestingly, Imbalance of the RAAS and direct effect of DPP4 promote vascular system damage, which result diabetic patients might be more affected by COVID-19, However, the interaction mechanism between DPP4 and RAAS (including ACE2) has not clear (9).

In the context of the COVID-19 pandemic, more attention should be paid to patients with cancer (10). One clinical study collated and analyzed 641 COVID-19 cases from 14 hospitals in Hubei Province, China. A more severe prognosis and higher mortality due to COVID-19 are seen in patients with cancer than in patients without cancer. This may be due to the low immune function of patients with cancer, which makes these patients more vulnerable to infection (11). A cohort study reporting data from the COVID-19 and Cancer Consortium Registry database showed that breast cancer is the most common type of cancer among 928 COVID-19 patients (12). In another meta-analysis, COVID-19 patients with breast cancer accounts for 13% after lung cancer (24.7%) and colorectal cancer(20.5%) (13).Treatment for COVID-19 has not yet been established, and breast cancer patients are a particularly fragile population that requires effective treatment to manage COVID-19. This review aimed to discuss and evaluate the effect of estrogens, estrogen receptors (ERs), and ER modulators on managing COVID-19 in patients with breast cancer.

## Estrogen Receptors: Structure and Isoforms

ER is a ligand-activated transcription factor that belongs to the steroid and nuclear hormone receptor superfamily (14). ER is closely associated with aberrant proliferation, inflammation, and development of breast cancer (15, 16). ER is classified into ERα and ERβ subtypes, encoded by *ESR1* and *ESR2* located on chromosomes 6q25.1 and 14q23.2, respectively (17, 18). The structure of the full-length product of ERα transcripts is divided into several functional domains as follows: The N-terminal domain, the DNA-binding domain (DBD), the ligand-binding domain (LBD), and two activation domains (AF1 and AF2) (19). Hormonal therapies are initially effective because they are dependent on the activation of ERα by estrogen.

**Figure 1:**
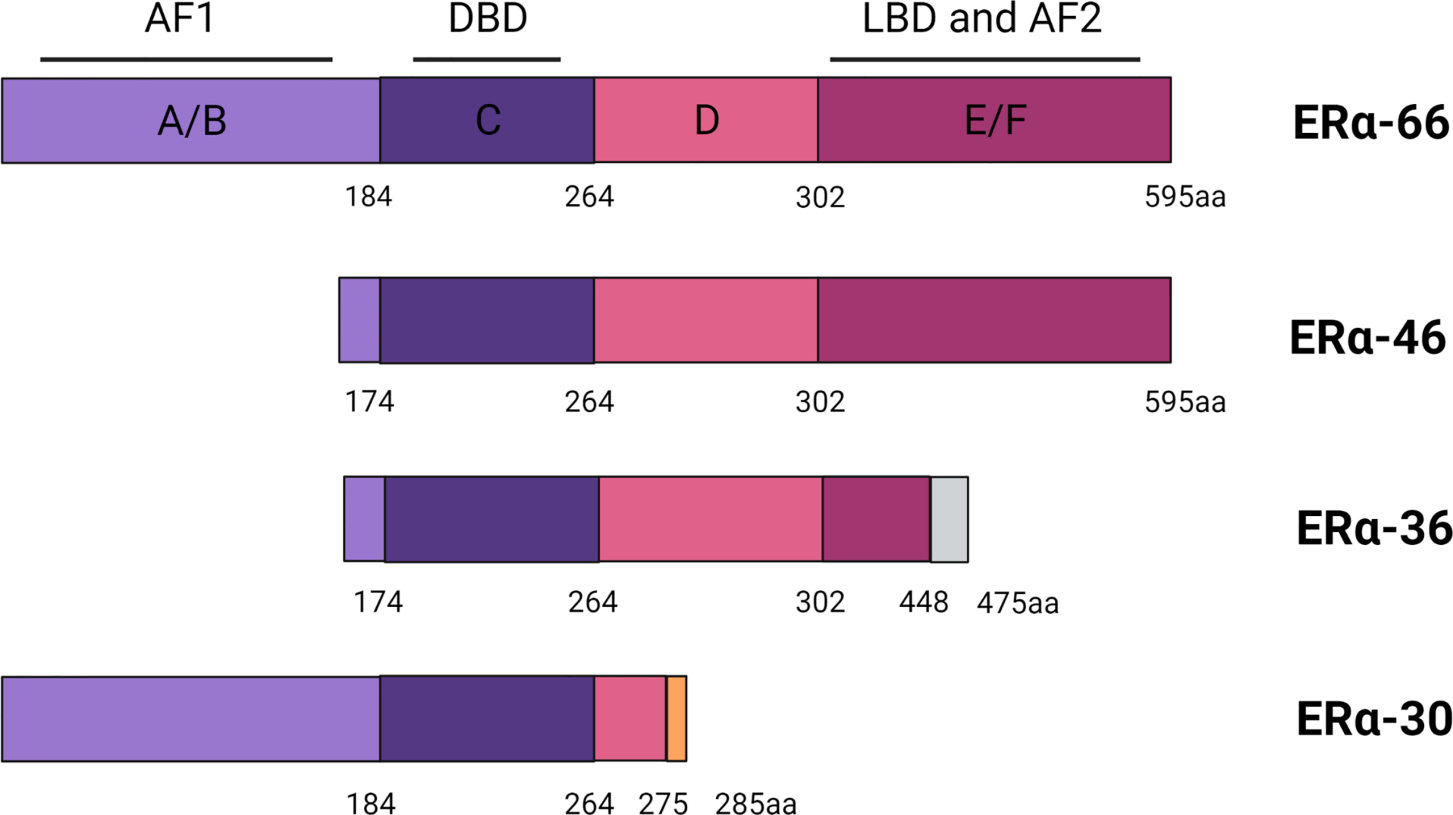
Scheme of ERα, ERα-46, ERα-36 and ERα-30 structure.

Various truncated shorter isoforms of ERα have been discovered over the past 20 years, among which ERα-46 and ERα-36 are known best. ERα-46 lacks the first 173 amino acids in the N-terminus of ERα-66, is transcribed under the control of promoters E and F, and contains complete exons 2–8, lacking the AF1 transactivation domain (19). ERα-46 can decrease the response to E2 and ERα-46 expression, and the size of ERα^+^ tumors is negatively correlated with its expression (19, 20). ERα-36 is a typical ERα-66 truncated isomer, which is transcribed from an unknown promoter located in the first intron of *ESR1* and encoded by exons 2–6 and 9. Compared with ERα-66, ERα-36 lacks the transcription activation domains AF1 and AF2, but still retains the DBD, hinge domain, and LBD, and contains a new structural domain composed of 27 amino acids at the C-terminal (21). This specific C-terminal domain amino acid sequence of ERα36 plays an important role in the interaction between ERα36 and p-ERK2, and may also change the LBD, resulting in different binding affinities (22). Recently, a new 30-kDa ERα variant was identified, called ERα-30. ERα-30 is encoded by complete exons 1–3 and 8 and partial exons 4 and 6. ERα-30 is different from ERα-66. ER-α30 has a partial hinge domain and lacks the C-terminal LBD/AF2 transactivation domain, but has complete AF1 and DBD, with a specific domain composed of 10 amino acids at the C-terminal. Furthermore, this structural difference may also cause ERα-30 to induce a distinctly different set of transcriptional procedures (23). However, there are still no clinical data from trials considering ERα-30 expression in breast cancer patient (**Figure 1**).

## Endocrine Therapy and Breast Cancer

Breast cancer is the most frequently diagnosed cancer among women worldwide, and nearly 630,000 patients with breast cancer died in 2018 (24, 25). Over 90% of patients are diagnosed with early-stage breast cancer, and approximately 70% of them are ER-positive (ER^+^) (26). Anti-estrogen therapy was the first effective targeted therapy for ER^+^ breast cancer and has now become the main adjuvant therapy for ER^+^ patients (27). The therapeutic effect of this treatment is due to the blockade of estrogen receptors or inhibition of estrogen production (28, 29).

**Figure 2:**
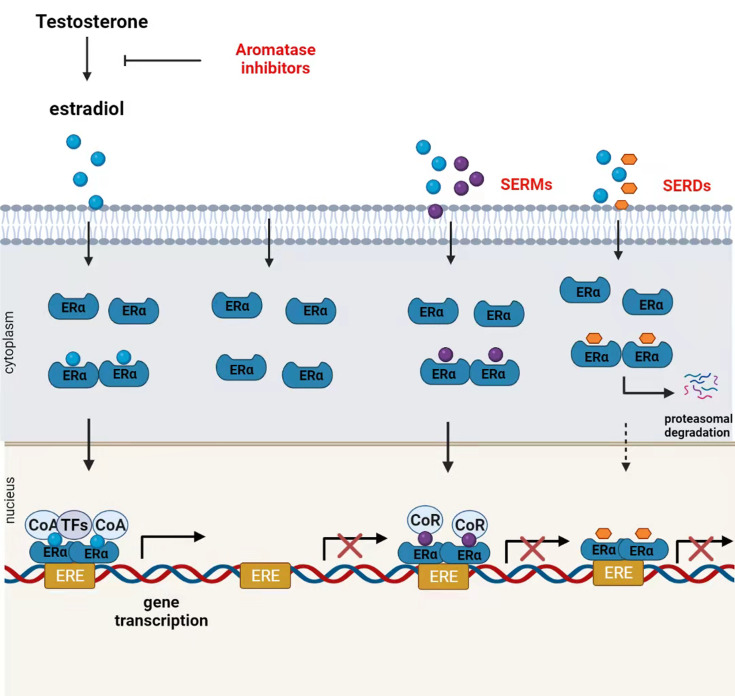
Mechanism of action of endocrine therapies. Ovaries, the adrenal gland, and other organizations produce testosterone that is transformed into estradiol by aromatase. In the presence of circulating estrogen, estrogen receptor (ER)α undergoes conformational changes, form homo- or heterodimers and then migrates to the nucleus, where ERα dimers bind coactivators (CoA) to form a transcriptionally active ERα complex. ERα complex can regulate the transcription and activation of various genes by binding to the estrogen response element (ERE)-encoding gene or interacting with other transcription factors. Aromatase inhibitors block estrogen production by inhibiting androgen conversion to estrogens. Selective estrogen receptor modulators (SERMs) competitively inhibit the binding of estrogen to ERα. SERM-bound ER dimers bind to co-repressors (CoR) inhibiting ER transcriptional activity in breast cancer tissues. Selective estrogen receptor downregulators (SERDs) downregulate the receptor protein expression by inducing ER degradation.

Selective ER modulators (SERMs), selective ER downregulators (SERDs), and aromatase inhibitors (AIs) are approved for endocrine therapy in patients with ER^+^ breast cancer (30). SERMs act primarily at the receptor level to compete with estrogen for the activation of ERα (31). For example, tamoxifen (TAM), the first molecular targeted therapy for breast cancer, can reduce the 10-year recurrence rate and corresponding mortality. TAM is still widely used in premenopausal patients with ER^+^ breast cancer (32). Fulvestrant is a SERD that mainly inhibits ERα dimerization and induces ER degradation to downregulate ERα levels (33). Recently, Guan et al. showed that fulvestrant-like antagonists inhibit ER transcriptional activity mainly by slowing down the mobility of ER in the nucleus (34). AIs such as letrozole, anastrozole, and exemestane decrease systemic estrogen levels by blocking the conversion of testosterone to estrogen (35). Although TAM has been the standard endocrine therapy for ER^+^ patients for decades, AIs have shown better efficacy than TAM (36). Studies have shown that exemestane combined with ovarian suppression significantly reduces the recurrence rate in premenopausal patients compared to that seen with TAM combined with ovarian inhibition (37). Although most patients initially receive ERα-targeted hormone therapy, after an average of 5–20 years, up to 20% of ERα^+^ patients develop metastatic lesions (38) (**Figure 2**).

The COVID-19 pandemic has changed the breast cancer treatment approach. The Society of Surgical Oncology and American Society of Breast Surgeons specifically recommend elective surgery for patients with ER^+^ breast cancer and neoadjuvant endocrine therapy (NET) as a safe alternative to traditional “surgical priority” (39, 40). A randomized clinical trial showed that NET successfully improved the surgical decision from mastectomy to breast-conserving surgery in approximately 80% of patients with ER^+^ breast cancer, with AIs showing greater efficacy than TAM (41, 42). Interestingly, a recent study showed that patients with COVID-19 and breast cancer may benefit from anti-estrogen or TAM-based therapies (43).

## ER and SARS-CoV-2 Infection Susceptibility

Published global COVID-19 epidemiological data show that a higher risk of both infection and death is seen in men than in women (44). Similarly, a previous study showed that male mice were more likely to be infected with SARS-CoV than female mice at the same age, and the difference was more obvious with an increase in age (45). We speculate that hormonal, genetic, and behavioral contribute to the observed gender differences, although there is no clear experimental data (46). This sex difference may be due to the decrease in ACE2 activity by estradiol, which is independent of sex chromosome complement (47). A clinical study conducted in Wuhan showed that non-menopausal female patients with COVID-19 had milder severity, better prognosis, and shorter hospital stays than in those going through menopause, suggesting that menopause is an independent risk factor for female patients with COVID-19 (48).

The immune difference between men and women also leads to divergence between males and females in response to SARS-CoV-2. Women have a stronger innate immune system than men, which provides rapid and extensive protection against viral infections (49). In the enrichment analysis of normal and SARS-CoV-2 infected human tissues, it was found that the expression levels of ERα and ERβ were positively correlated with the enrichment of immune cells (50). Estrogen has a marked effect on innate and acquired immune responses, and ERs are expressed in a variety of cell types in various tissues, including the immune system (51). ERs change the activity of immune cell types related to the immune response by regulating cells and pathways in the innate and adaptive immune systems (52). Estradiol has a dual effect: at low concentrations, it plays a pro-inflammatory role, and at high concentrations, it plays an anti-inflammatory role. In particular, estrogen inhibits the expression of pro-inflammatory IL-6 by directly affecting CD16 expression. In particular, estrogen suppresses the expression of pro-inflammatory IL-6 and the production of IL-12 from stimulated macrophages by directly changing the expression of CD16 (49, 52). Both estrogen and ERα contribute to the activation and proliferation of T-lymphocytes and lead to high expression of IFN-γ. Researchers have shown that IFN-β and IFN-γ can effectively inhibit the replication of SARS-CoV, and the combination of IFN-γ and IFN-γ can enhance the anti-SARS-CoV effect. This evidence also supports that estrogen and ERs may be related to SARS-CoV-2 infection (51).

Interestingly, experimental studies in female mice infected with SARS-CoV showed that ovariectomy or treatment with estrogen receptor antagonist in female mice increased death rate, which indicate estrogen receptor play a protective effect in mice infected with SARS-CoV and SERMs may decrease females’ mortality (53). The estrogen/ER axis may be a potential target for the treatment of viral infections, especially for patients with breast cancer.

## Effect of SERMs on SARS-CoV-2 Infection in Breast Cancer

ER overexpression plays a critical role in inhibiting viral replication. SERMs inhibit viral replication through non-classical pathways associated with ER. Toremifene, the first generation of nonsteroidal SERMs, demonstrates latent effects in blocking various virus infections, including SARS-CoV, Middle East respiratory syndrome-CoV, and Ebola infections (54). A previous study showed that toremifene may inhibit the S glycoprotein *via* perturbation of the fusion core and eventually inhibit SARS-CoV-2 replication (55). Bazedoxifene can interact with SARS-CoV-2 main protease, and the effect of raloxifene is *via* the attenuation of the combination of SARS-CoV-2 with its target cells (56, 57). Moreover, a study showed that bazedoxifene and raloxifene can inhibit interleukin (IL)-6 signaling to prevent a cytokine storm and ARDS and reduce mortality in patients with severe COVID-19 (58). Clomiphene is primarily used to treat female infertility due to anovulation and acts as both an estrogenic agonist and antagonist (59). Clomiphene may inhibit SARS-CoV-2 entry by impairing endosome/lysosome function (60) **(** **Table 1**).

**Table 1:** Effect of SERMs on the biology of SARS−CoV−2.

Drug name	Chemical structure	Pharmacological action	Functional Effects	References
Toremifene	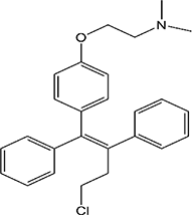	Antiestrogen/Estrogen agonist	Inhibition of virus replication and blocking of virus entry	(54)
Raloxifene	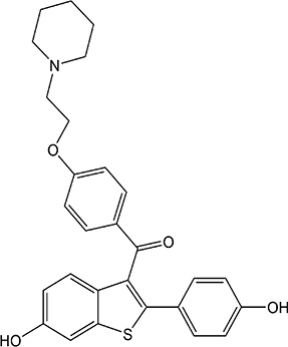	Antiestrogen/Estrogen agonist	blocking of virus entry and inhibit IL-6 signaling	(56–58)
Bazedoxifene	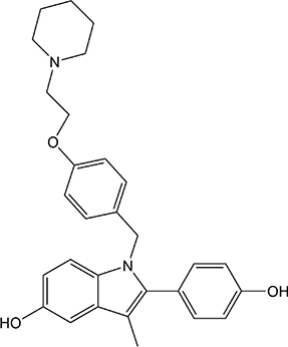	Antiestrogen/Estrogen agonist	Interaction with SARS−CoV−2 main protease and inhibit IL-6 signaling	(56–58)
Clomiphene	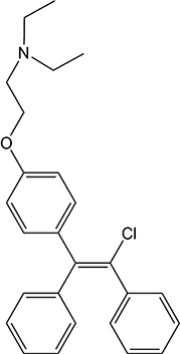	analogue	Impair ate endosome/lysosome function	(60)
Tamoxifen	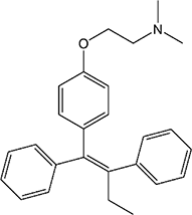	Antiestrogen/Estrogen agonist	bind to AR and inhibit its activity	(61)

### Raloxifene

Raloxifene is a second-generation selective benzothiophene SERM with agonist or antagonist activity on estrogen. Raloxifene pass through the cytoplasmic and the nuclear membrane to reach nucleus, then in which the benzothiophene ring binds to ER and has a similar affinity with E2. The drug is FDA-approved used to treat and prevention of osteoporosis in postmenopausal women, and reduction of the risk of invasive breast cancer in postmenopausal women (62, 63). Recently, raloxifene has been selected as a clinical candidate against SARS-CoV-2 by using an integrated approach between the EXSCALATE platform and predicted the high probability of the drug to interact with several relevant SARS-CoV-2 by the virtual screening protocols (64). Raloxifene demonstrates an *in vitro* antiviral activity, in terms of inhibition of viral replication and/or infection, against EBOV, influenza A, and hepatitis C viruses (HCV) (65, 66).

The possible mechanism of action of raloxifene in viral infections is directly related to its activity through modulation of ER and the related pathways (67). The young adult men appear more severe clinical outcome in HCV progresses than women. And a randomized trial demonstrate that early menopause women is associated with reduced treatment efficacy and accelerated progression of HCV-associated liver fibrosis (67, 68). Moreover, raloxifene showed efficacy in nasal epithelial cells isolated from female patients infected with influenza A virus (69). Another possible mechanism is that raloxifene through direct targeting of viral life cycle (70). Compared with other SERMs, raloxifene show a low oral bioavailability. But raloxifene has higher pulmonary distribution and exerts its pharmacological activity at very low circulating levels. Although the safety profile of raloxifene in the treatment of COVID-19 is not yet confirmed, raloxifene treatment for 22 years in postmenopausal women and not found additional safety risks (64). Raloxifene is effective in preventing osteoporosis in menopausal women. It is also found to be effective in preventing breast cancer. Compared with tamoxifen, raloxifene does not increase the risk of endometrial cancer and cardiovascular disease. In view of its potency against COVID-19, raloxifene is a promising option for the treatment of breast cancer with SARS-CoV-2 infection.

### Tamoxifen

Tamoxifen, first-generation SERM, has been found effective against HIV, HCV, and herpes simplex virus 1 (HSV-1) (65). Tamoxifen suppresses HCV genome replication by eliminating RNA polymerase NS5B -replication complex (RC) association which is functionally regulated by the ERα (68). In HIV vitro studies, tamoxifen interferes with HIV replication by inhibiting the ability of HIV-promoter-driven transactivation in monocytes and CD4 + T lymphocytes induced by phorbol myristate acetate (the protein kinase C activator) (71). Recently, a study showed that tamoxifen and clomiphene may inhibit the production of SARS-CoV-2 S protein by impeding viral entry (72).

The majority of breast cancer often express androgen receptor(AR), with 84%to 95% in ER+ breast cancer, 50% to 63% in ER−/HER2+ and 10% to 53% in triple negative breast cancer (TNBC) (73). Emerging clinical data show the downregulation of TMPRSS2 and ACE2 expression by the androgen receptor (AR) antagonist GT0918 in prostate and lung cells. TMPRSS2 is controlled by androgen receptor (AR) signaling and is considered a requirement for the prime SARS-CoV-2 spike protein for entry into target cells. These results suggest that SARS-CoV-2 infection is likely to be androgen-mediated (74, 75). Therefore, AR inhibitors like nonsteroidal antiandrogen (enzalutamide, bicalutamide, apalutamide, and darolutamide, steroidogenesis inhibitors, 5-alpha reductase inhibitors, and chemical castration with gonadotropin-releasing hormone analogs could be valid treatment in the COVID-19 patients (76). Interestingly, the use of high-dose estrogens may be an attractive way to limit the growth and spreading of prostatic cancer cells (77). The interplay of ER and AR would suggest in this breast cancer patients that would likely benefit from both the antitumor and the anti-COVID-19 effects. Based on this rationale, TAM can directly bind to the AR and inhibit its activity (61). The clinical trial NCT04353180 is testing the tamoxifen in combination with isotretinoin and trimethoprim in COVID-19 patients, but the specific efficacy is not clear (43). Unlike TAM, AIs can inhibit the transformation of androgen to estrogen. In patients receiving this treatment, androgen accumulation may increase the expression of TMPRSS2 in tissues through AR signal activation, resulting in increased susceptibility to SARS-CoV-2. Experimental results show that estrogen upregulates the expression of TMPRSS2 through the ERb2/Src-IGF-1R/NFκB pathway, which means that AIs may inhibit the production of estrogen and subsequent TMPRSS2 expression through this pathway rather than androgen/AR signaling, to promote the upregulation of TMPRSS2 and exert potent anti-COVID-19 activity (36, 78). In fact, Tamoxifen can increase the pH value of lysosomes and change the kinetics of endosomes, thereby potentially interfere with the invasion of SARS-CoV-2 (79).

A recent clinical study on a group of patients with breast cancer who subsequently contracted SARS-CoV-2, excluding patients with potential risk factors, showed that 14 females treated with TAM were not as sensitive to infection compared to non-treated patients, and the effect of therapy was elevated in postmenopausal women. But more clinical trials are needed to prove its effectiveness due to the small sample size (56). These results suggest that short-term use of TAM may help to treat patients with breast cancer and COVID-19, and long-term use of TAM may decrease susceptibility to SARS-CoV-2. This may be due to the reduction of ER expression after long-term use of TAM (80). Studies have shown that there is also a large number of ERs in the lung tissue, and TAM may have an apoptotic effect through ER in the lung tissue (81, 82). TAM is a recognized inhibitor of P- glycoprotein, which is not affected by ERs, and it inhibits T-lymphocyte function and interferon (IFN) release. Long-term use of TAM may increase the risk of COVID-19 due to its anti- estrogen and P-glycoprotein inhibitory effects (83).

## Conclusions

The current COVID-19 pandemic has emphasized the importance of developing anti-viral drugs. SERMs, such as Tamoxifen has been selected as a clinical candidate for clinical studies in COVID-19 patients. Because malignant breast tumors often express AR, some literature evidence showed that combination of tamoxifen and anti-AR therapies could be a potential therapeutic strategy for patients with breast cancer and SARS-CoV-2 infection. However, Venous thromboembolism (VTE) is one of the important side effects of Tamoxifen and VTE was frequently found in COVID 19 infection. This is a huge challenge for COVID 19 patients with breast cancer using Tamoxifen. Although some researchers think that SERMs can be beneficial in patients with COVID 19, and its safety and validity of in the treatment of COVID-19 is not yet known. We need more clinical study to careful monitoring of potential cardiovascular and thromboembolic risks in the future.

## Author Contributions

JQ and JC for research project with conception, organization, and execution. SH, FY, LN, and YW for statistical analysis with design, execution, review and critique. JQ and JC for manuscript preparation with writing of the first draft, review and critique. All authors contributed to the article and approved the submitted version.

## Funding

This research is respectively supported by the National Natural Science Foundation of China (No.81973574, No.82174082, No.81860443), and the Natural Science Foundation of Guangxi (No.2019GXNSFFA245001).

## Conflict of Interest

The authors declare that the research was conducted in the absence of any commercial or financial relationships that could be construed as a potential conflict of interest.

## Publisher’s Note

All claims expressed in this article are solely those of the authors and do not necessarily represent those of their affiliated organizations, or those of the publisher, the editors and the reviewers. Any product that may be evaluated in this article, or claim that may be made by its manufacturer, is not guaranteed or endorsed by the publisher.
